# Patient perspectives about deployment of artificial intelligence decision support tools in a safety-net healthcare system

**DOI:** 10.1093/jamiaopen/ooag029

**Published:** 2026-03-17

**Authors:** Nicholas Phelps, Patrick Vossler, Avni Kothari, Katherine Steineman, Seth Goldman, Arturo Gasga, Susan Ehrlich, Hemal Kanzaria, Julia Adler-Milstein, Jean Feng, Paige Nong, Lucas S Zier

**Affiliations:** Department of Medicine, University of California San Francisco, San Francisco, CA 94122, United States; Department of Epidemiology and Biostatistics, University of California San Francisco, San Francisco, CA 94122, United States; Zuckerberg San Francisco General Hospital, San Francisco, CA 94110, United States; San Francisco Department of Public Health, San Francisco, CA 94102, United States; Department of Medicine, University of California San Francisco, San Francisco, CA 94122, United States; Zuckerberg San Francisco General Hospital, San Francisco, CA 94110, United States; San Francisco Department of Public Health, San Francisco, CA 94102, United States; Department of Medicine, University of California San Francisco, San Francisco, CA 94122, United States; Zuckerberg San Francisco General Hospital, San Francisco, CA 94110, United States; San Francisco Department of Public Health, San Francisco, CA 94102, United States; Department of Medicine, University of California San Francisco, San Francisco, CA 94122, United States; Zuckerberg San Francisco General Hospital, San Francisco, CA 94110, United States; San Francisco Department of Public Health, San Francisco, CA 94102, United States; Department of Medicine, University of California San Francisco, San Francisco, CA 94122, United States; Zuckerberg San Francisco General Hospital, San Francisco, CA 94110, United States; San Francisco Department of Public Health, San Francisco, CA 94102, United States; Zuckerberg San Francisco General Hospital, San Francisco, CA 94110, United States; San Francisco Department of Public Health, San Francisco, CA 94102, United States; Zuckerberg San Francisco General Hospital, San Francisco, CA 94110, United States; San Francisco Department of Public Health, San Francisco, CA 94102, United States; Department of Emergency Medicine, University of California San Francisco, San Francisco, CA 94122, United States; Department of Medicine, University of California San Francisco, San Francisco, CA 94122, United States; Department of Epidemiology and Biostatistics, University of California San Francisco, San Francisco, CA 94122, United States; Zuckerberg San Francisco General Hospital, San Francisco, CA 94110, United States; San Francisco Department of Public Health, San Francisco, CA 94102, United States; Division of Health Policy and Management, University of Minnesota Twin Cities, Minneapolis, MN 55455, United States; Department of Medicine, University of California San Francisco, San Francisco, CA 94122, United States; Zuckerberg San Francisco General Hospital, San Francisco, CA 94110, United States; San Francisco Department of Public Health, San Francisco, CA 94102, United States

**Keywords:** artificial intelligence, predictive methods, generative artificial intelligence, patient attitudes

## Abstract

**Objectives:**

To assess patient awareness, trust, perceived benefits, and risks of artificial intelligence (AI) in clinical care within an urban safety-net health system.

**Materials and Methods:**

We surveyed 313 patients from November 2024 to January 2025 regarding AI awareness, trust in AI-assisted decision-making, and preferences for transparency and oversight. Quantitative analyses assessed associations between AI awareness and perceived benefit; qualitative analysis identified themes influencing trust.

**Results:**

While 84% were familiar with commercial AI, fewer than half recognized the use of AI in medical decision support. Greater AI awareness was associated with higher perceived benefit (all *P* < .001). Participants emphasized transparency (92%), clinician oversight (82%), and validation as critical to trust.

**Discussion:**

This study provides one of the first assessments of patient perspectives on AI within a safety-net healthcare setting. Patients view clinical AI favorably but demand transparency and clinician involvement.

**Conclusions:**

Patient education and engagement are essential for equitable, trustworthy AI deployment.

## Introduction

Artificial intelligence (AI) holds immense promise to improve health outcomes and advance equity, particularly in resource-limited healthcare settings.^1^ Safety-net healthcare systems, which serve vulnerable populations, are increasingly adopting AI-driven decision-support tools to enhance clinical care. However, little is known about how patients perceive the deployment of these technologies, including their expectations, concerns, and trust in AI-assisted decision-making.^1^^,^^2^ While previous work has examined clinician perspectives,^3^^,^^4^^,^^5^ patient voices remain underrepresented in discussions about AI governance, particularly within marginalized communities. We sought to assess patient perceptions of AI within our urban safety-net healthcare system. Specifically, we explored patient awareness of AI applications in healthcare, their trust in AI-assisted recommendations, and their expectations regarding transparency, clinician oversight, and performance validation. As AI becomes more integrated into clinical workflows, patient education may play a pivotal role in shaping trust and adoption. Here, we present the results of a survey designed to inform strategies for responsible AI deployment in the safety-net setting.

## Methods

From November 2024 to January 2025, we distributed an electronic survey to patients at an urban safety-net healthcare system. The survey included a validated technology literacy assessment,^6^ questions on AI in healthcare, and 3 AI-enabled decision-support scenarios: predictive AI for acute illness, predictive AI for chronic illness, and generative AI for chronic illness management. Descriptive statistics summarized responses. A chi-square test compared perceived accuracy and trust between predictive and generative AI. Exploratory factor analysis revealed distinct patterns in AI awareness, with factor loadings suggesting that participants’ familiarity with AI applications clustered into separate dimensions (general commercial AI, predictive clinical AI, and generative clinical AI). Participants were therefore stratified by their overall AI awareness level to examine how familiarity with AI technologies mediated perceptions of accuracy and trust. Effect sizes were calculated using epsilon-squared (ε^2^) for the Kruskal–Wallis H-tests to quantify the magnitude of differences across AI awareness levels. The authors performed a thematic analysis of free-text responses using a codebook approach, with 3 members of the research team independently coding them. Discrepancies were reconciled in a synchronous meeting, and final themes were agreed upon by all 3 coders. These themes were operationalized in a structured prompt for LLM-assisted annotation using GPT-4o-mini via a HIPAA-compliant API (temperature = 0.0, fixed random seed). The LLM assigned the single most appropriate theme to each of 939 responses and provided a justification for each annotation; responses not fitting any theme were coded as “None.” Investigators reviewed all “None” annotations, which primarily consisted of brief non-substantive responses (eg, “I don’t know”). Theme counts were then tallied from the LLM annotations. Analysis was conducted in July 2025; additional technical details are provided in the Supplement.

## Results

A total of 313 individuals (response rate 5%) completed the survey. Responses were inverse weighted by survey completion rate to reflect the patient characteristics of the entire hospital. See Table 1 for patient characteristics of survey responders before and after reweighting of the data. Technology literacy was high, with 71% having high tech literacy. In contrast, there was a substantial difference in awareness between medical and non-medical AI uses: 84% were familiar with commercial applications, but fewer than half knew of clinical use in medicine—43% had heard of AI for disease diagnosis, 40% for predicting illness risk or mortality, and 47% for summarizing medical records. Participants were stratified into low and high AI awareness based on self-reported familiarity with AI in general and knowledge of specific AI applications.

**Table 1.** Characteristics of participants

Characteristic	Unweighted	Weighted	ZSFG population (%)
Age—no. (%)			
16-24	5 (2)	10 (2)	1
25-34	28 (9)	67 (11)	8
35-44	45 (15)	97 (16)	16
45-54	59 (19)	123 (20)	19
55-64	87 (28)	163 (26)	25
65-74	66 (21)	117 (19)	21
75-84	16 (5)	28 (5)	7
85-94	2 (1)	6 (1)	2
95+			0
Prefer not to say	2 (1)	4 (1)	0
Gender—no. (%)			
Male	152 (49)	297 (48)	45
Female	146 (47)	298 (48)	55
Transgender	4 (1)	7 (1)	
Nonbinary/gender-nonconforming	6 (2)	9 (1)	
Prefer not to say	2 (1)	4 (1)	0
Race/ethnicity—no. (%)			
American Indian or Alaska Native	15 (5)	30 (5)	1
Asian	35 (11)	92 (15)	17
Black or African American	44 (14)	77 (13)	11
Native Hawaiian or Other Pacific Islander	5 (2)	14 (2)	1
White	151 (49)	244 (40)	22
Other	94 (30)	224 (36)	5
Hispanic	72 (23)	182 (30)	42
Decline to answer			1
Race not specified			0

**Figure 1.**
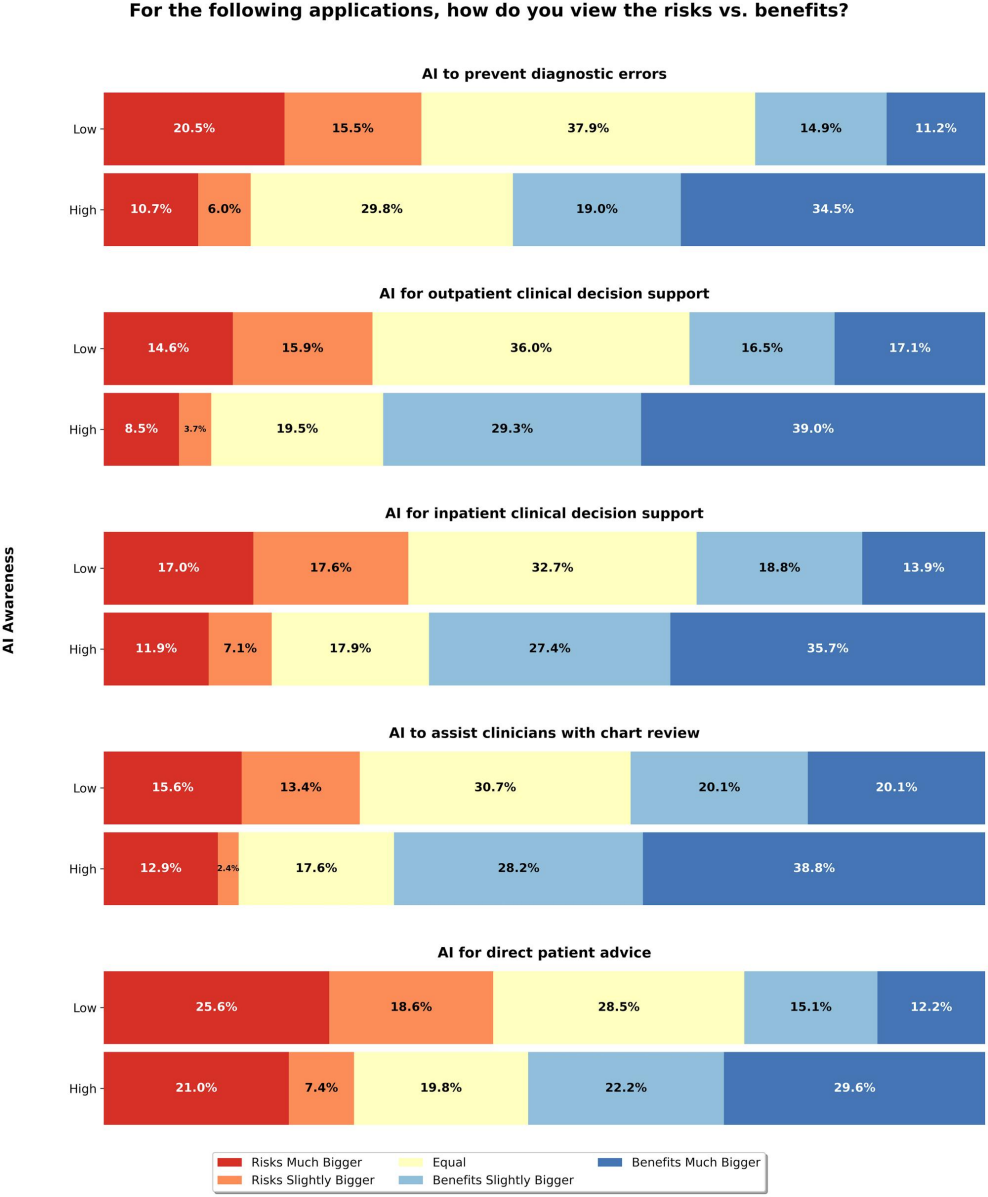
Patient perceptions of the risks vs benefits of the 5 clinical AI use cases.

AI transparency was a dominant concern, with 92% wanting notification when AI tools are used in their clinical care. Additionally, 82% emphasized “human-in-the-loop” systems, advocating for clinician oversight. When asked about different clinical AI use cases, participants broadly felt that the potential benefits of AI use outweighed its risks. This trend was most pronounced amongst participants with high AI awareness. When presented with specific clinical scenarios, approximately 64% of participants indicated that they would trust AI-generated recommendations given to their doctors, whether for predictive purposes (such as preventing hospital readmissions or detecting early sepsis) or for treatment guidance (such as adjusting diabetes medications or addressing substance use). When asked about risks versus benefits, participants with high AI awareness consistently perceived greater benefits relative to risks across all clinical scenarios (Kruskal–Wallis H-test, all *P* < .001). Effect sizes demonstrated medium to large associations between AI awareness and benefit perception, with the strongest effects observed for AI in outpatient care (ε^2^ = 0.178) and overall healthcare applications (ε^2^ = 0.155). Medium effects were found for AI in hospital settings (ε^2^ = 0.133), medical records (ε^2^ = 0.088), and patient advice (ε^2^ = 0.095). Figure 1 shows perceptions of risk vs benefit of each clinical AI use case, stratified by AI awareness. Stratification by gender, age, and race/ethnicity revealed significant but smaller associations with risk-benefit perceptions compared to AI awareness (Table S1). Gender showed the strongest demographic effect, with men perceiving greater benefits than women across most scenarios.

**Figure 2.**
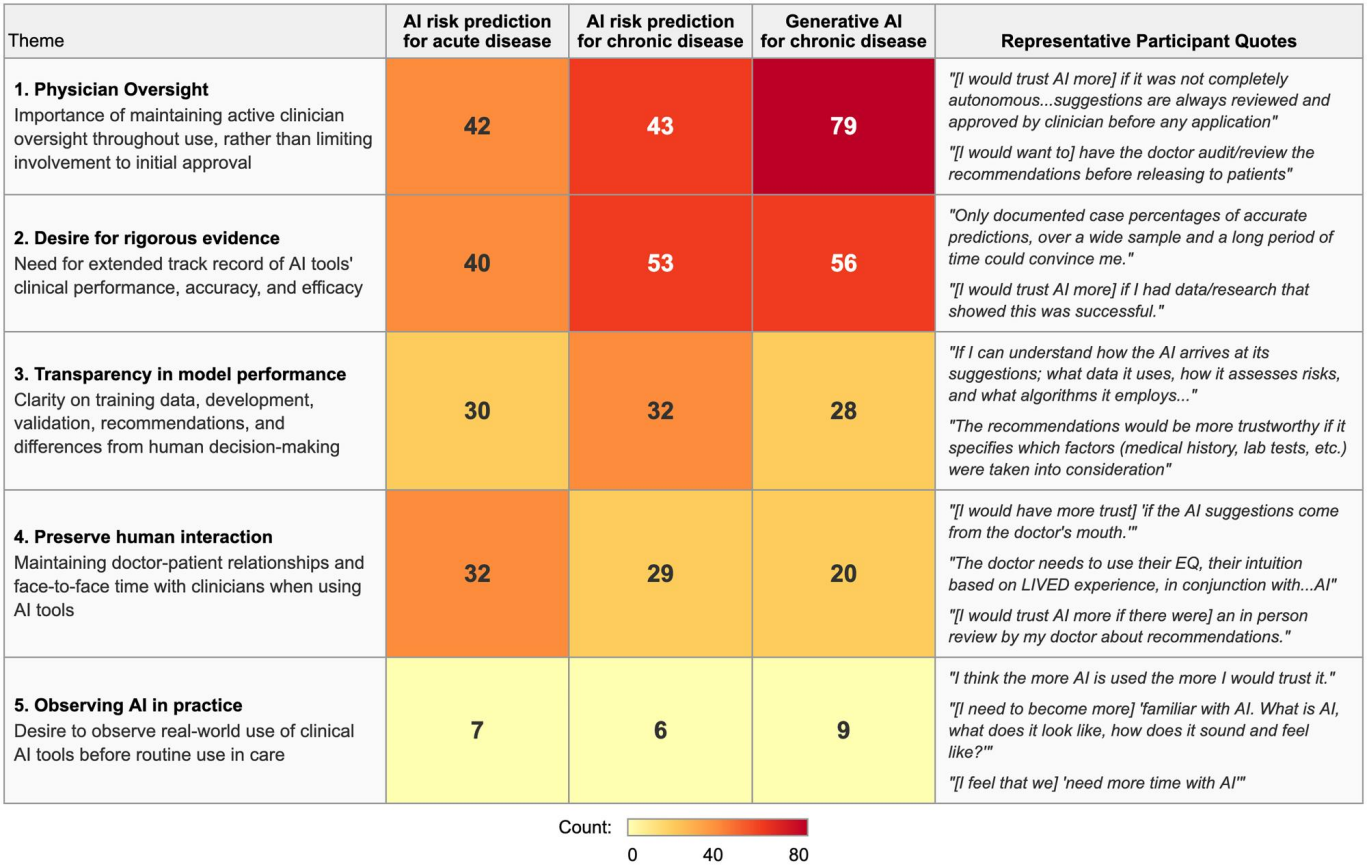
Qualitative themes (y-axis) underlying participants’ trust in specific clinical AI use cases (x-axis): boxed numbers represent the incidence of each theme in participants’ free-text responses regarding their trust in each specific use case.

Qualitative analysis identified 5 themes shaping trust in AI deployment: (1) the necessity of physician oversight, (2) demands for transparency in model performance and safety, (3) a preference to preserve human interaction in AI-assisted care, (4) interest in observing use of AI tools in clinical practice, and (5) desire for rigorous evidence of AI tools’ clinical performance (see Figure 2 for a description of themes and direct quotations). Figure 2 shows the incidence of each trust theme by AI clinical scenario queried. Physician oversight and rigorous evidence were the most cited themes, particularly for generative AI in chronic disease management and AI risk prediction for chronic disease.

## Discussion

This study provides one of the first assessments of patient perspectives on AI in clinical medicine within a safety-net healthcare setting. Participants were asked about real-world clinical applications of AI currently in use within our health system. Despite high general technology literacy, patient awareness of AI’s clinical applications lagged behind familiarity with commercial AI. Participants were asked about real-world clinical applications of AI (including some currently in use within our health system); results are therefore applicable to the iterative improvement of these tools as well as the deployment of similar tools in the future. Participants emphasized transparency, clinician oversight, and robust validation as critical factors for fostering trust in deployed decision support tools.

While we hypothesized that patient perspectives on AI in safety-net settings might reveal distinct concerns due to unique patient populations and resource constraints, our findings largely mirror those from general populations regarding the importance of transparency, clinician oversight, and validation for fostering trust.^2^^,^^3^^,^^7^^,^^8^ This suggests that foundational trust principles are broadly maintained amongst disparate patient populations and practice settings, yet their effective implementation in safety-net communities demands reinforced education and engagement to ensure equitable adoption and address existing disparities.

Notably, participants with greater AI awareness were more likely to perceive the benefits of AI as outweighing the risks, underscoring a potential role for patient education in **fostering trust in the use of these tools.** Given that AI-driven decision support is increasingly shaping patient care, efforts to educate patients on AI’s capabilities, limitations, and validation processes could improve acceptance and trust.

This study has several important limitations. The low 5% response rate, despite inverse weighting to align with hospital demographics, introduces the potential for non-response bias and may limit the generalizability of our findings. Moreover, while weighting mitigated initial imbalances, our sample still exhibited an overrepresentation of White participants and an underrepresentation of Hispanic participants, suggesting that more targeted recruitment strategies are needed in future research to achieve truly representative patient populations.

These findings highlight the need for patient voices to be actively incorporated into AI policy and governance frameworks, particularly in safety-net settings where AI tools may play a crucial role in addressing healthcare disparities. Patients in our study asked reasonable and insightful questions about AI performance and validation, suggesting a need for ongoing engagement between healthcare institutions and the communities they serve. Our survey results are now being used to inform AI deployment strategies within our health system, with a focus on transparency, education, and patient-centered oversight. As AI continues to evolve in clinical medicine, ensuring that patient perspectives shape its implementation will be essential for fostering trust and equitable adoption.

## Supplementary Material

ooag029_Supplementary_Data

## Data Availability

The data underlying this article cannot be shared publicly, to maintain the privacy of individual subjects. The data will be shared on reasonable request to the corresponding author.
