# Supplementary material for: Patient perspectives about deployment of artificial intelligence decision support tools in a safety-net healthcare system
Source: JAMIA Open. 2026 Mar 17;9(2):ooag029. doi: 10.1093/jamiaopen/ooag029 (PMC12994689; doi:10.1093/jamiaopen/ooag029)
Supplement: ooag029_Supplementary_Data [file ooag029_supplementary_data.docx]

Supplementary Materials

# S1. LLM-Assisted Thematic Analysis: Technical Details

## Model Configuration

**Model:** GPT-4o-mini (gpt-4o-mini-2024-07-18) accessed via HIPAA-compliant API
**Temperature:** 0.0
**Seed:** 0
**Date of analysis:** July 2025

## Annotation Prompt

You are a qualitative researcher assisting in analyzing survey responses from patients at a safety net hospital regarding their attitudes towards artificial intelligence (AI) in healthcare. The survey includes free-response questions, and your task is to annotate each response with one of the following themes based on its content. These themes capture key concerns, expectations, and attitudes towards the use of AI in clinical settings:

Physician Oversight: Participants emphasized the importance of maintaining active and ongoing clinician oversight throughout the use of these tools, rather than limiting clinician involvement to an initial approval or sign-off.

Transparency in Model Performance and Safety: Participants wanted to be informed about the use of AI in their care. They also wanted clarity on aspects such as the data used to train models, how models were developed and validated, their specific recommendations, and how these differ from human decision-making.

Preference to Preserve Human Interaction in AI-assisted Care: Participants emphasized the importance of maintaining doctor-patient relationships and interaction, and expressed concerns about losing face to face time with clinicians when using AI tools.

Interest in Observing Use of AI tools in Clinical Practice: Participants expressed a desire to observe the real world use of clinical AI tools in practice before they felt comfortable with their routine use in clinical care.

Desire for Rigorous Evidence of AI tools' Clinical Performance: Participants expressed concerns about the lack of evidence regarding the clinical performance of AI tools including risk prediction models, and wanted to see an extended track record of accuracy and efficacy.

If a response does not fit any of these themes, annotate it as None.

Question asked: {question}
Patient response: "{response}"

Your task: Analyze the response and annotate it with the most appropriate theme from the list above. Provide a brief explanation for your choice to justify your annotation. Format your answer in JSON as follows:
{"theme": "<ANNOTATED THEME>", "explanation": "<YOUR REASONING FOR CHOOSING THIS THEME>"}

## Quality Control Procedures

**1. Theme Development:** Three members of the research team independently coded free-text responses. Discrepancies were reconciled in synchronous meetings until consensus was reached on five final themes, which were then operationalized for LLM annotation.

**2. Justification Requirement:** The LLM was required to provide an explanation for each theme assignment, enabling review of reasoning.

**3. Human Review:** Investigators reviewed all responses assigned "None" (no theme). These primarily consisted of brief non-substantive responses (e.g., "I don't know," "Same as previous," one-word answers).

**4. Reproducibility:** Deterministic model settings (temperature=0.0, seed=0) ensure identical outputs upon re-execution.

# S2. Risk-Benefit Perceptions by Demographic Characteristics

Weighted Kruskal-Wallis H-test results examining associations between demographic characteristics and perceptions of risks vs. benefits for five clinical AI use cases.

| **Demographic** | **Outcome** | **Sample Sizes** | **H** | **p (Bonferroni)** | **ε²** | **Sig.** |
| --- | --- | --- | --- | --- | --- | --- |
| Gender | AI to prevent diagnostic errors | Female: 112; Male: 123; Other: 10† | 23.67 | 0.0001 | 0.097 | Yes |
| Gender | AI for outpatient decision support | Female: 113; Male: 123; Other: 10† | 26.28 | 0.0000 | 0.107 | Yes |
| Gender | AI for inpatient decision support | Female: 115; Male: 124; Other: 10† | 36.25 | 0.0000 | 0.146 | Yes |
| Gender | AI to assist with chart review | Female: 119; Male: 134; Other: 11† | 17.00 | 0.0030 | 0.065 | Yes |
| Gender | AI for direct patient advice | Female: 118; Male: 124; Other: 11† | 10.35 | 0.0848 | 0.041 | NS |
| Age | AI to prevent diagnostic errors | 16-34: 25; 35-44: 33; 45-54: 44; 55-64: 69; 65-74: 55; 75+: 17† | 9.44 | 1.0000 | 0.039 | NS |
| Age | AI for outpatient decision support | 16-34: 24; 35-44: 37; 45-54: 46; 55-64: 66; 65-74: 54; 75+: 18† | 11.72 | 0.5816 | 0.048 | NS |
| Age | AI for inpatient decision support | 16-34: 26; 35-44: 33; 45-54: 47; 55-64: 72; 65-74: 53; 75+: 17† | 22.86 | 0.0054 | 0.093 | Yes |
| Age | AI to assist with chart review | 16-34: 25; 35-44: 35; 45-54: 50; 55-64: 74; 65-74: 60; 75+: 18† | 2.36 | 1.0000 | 0.009 | NS |
| Age | AI for direct patient advice | 16-34: 25; 35-44: 38; 45-54: 47; 55-64: 69; 65-74: 55; 75+: 17† | 10.35 | 0.9893 | 0.041 | NS |
| Race/Ethnicity | AI to prevent diagnostic errors | AIAN: 10†; API: 30; Black: 29; Hispanic: 48; Other: 14†; White: 114 | 6.05 | 1.0000 | 0.025 | NS |
| Race/Ethnicity | AI for outpatient decision support | AIAN: 11†; API: 31; Black: 30; Hispanic: 50; Other: 13†; White: 111 | 23.24 | 0.0046 | 0.095 | Yes |
| Race/Ethnicity | AI for inpatient decision support | AIAN: 11†; API: 32; Black: 31; Hispanic: 49; Other: 12†; White: 114 | 20.63 | 0.0142 | 0.083 | Yes |
| Race/Ethnicity | AI to assist with chart review | AIAN: 11†; API: 33; Black: 33; Hispanic: 52; Other: 14†; White: 121 | 13.47 | 0.2902 | 0.051 | NS |
| Race/Ethnicity | AI for direct patient advice | AIAN: 11†; API: 31; Black: 31; Hispanic: 52; Other: 14†; White: 114 | 8.00 | 1.0000 | 0.032 | NS |

**Notes:** Responses measured on 5-point scale: 1 = "risks much bigger than benefits" to 5 = "benefits much bigger than risks." "Don't Know" responses excluded. † indicates groups with n < 20. Bonferroni correction applied for 15 comparisons (adjusted α = 0.0033). NS = not significant. AIAN = American Indian/Alaska Native; API = Asian/Pacific Islander.

## Supplementary Narrative

To examine whether demographic characteristics were associated with risk vs. benefit perceptions, we conducted weighted Kruskal-Wallis H-tests comparing responses across gender (Male, Female, Other), age (16–34, 35–44, 45–54, 55–64, 65–74, 75+), and race/ethnicity (Hispanic/Latino, American Indian/Alaska Native, Asian/Pacific Islander, Black/African American, White, Other) groups for each of the five clinical AI use cases. A total of 15 tests were performed, with Bonferroni correction applied to control for multiple comparisons (adjusted α = 0.0033). Effect sizes were calculated using epsilon-squared (ε²).

After correction for multiple comparisons, 7 of 15 tests reached statistical significance. Gender was associated with risk-benefit perceptions for 4 of 5 outcomes (ε² = 0.04–0.15), with men perceiving greater benefits relative to risks compared to women across clinical AI applications. Age showed a significant association for 1 outcome (ε² = 0.01–0.09), with older participants perceiving greater benefits than younger participants. Race/ethnicity was associated with 2 outcomes (ε² = 0.03–0.10), with White participants perceiving greater benefits compared to Hispanic/Latino and other racial/ethnic minority groups.

These demographic effect sizes were generally smaller than those observed for AI awareness (ε² = 0.09–0.18), which showed consistent medium-to-large effects across all five outcomes. This supports the focus on AI awareness as the primary stratification variable in the main analysis, while acknowledging that demographic factors—particularly gender—also contribute to variation in risk-benefit perceptions.
